# Transcatheter Aortic Valve Replacement: a Kidney’s Perspective 

**DOI:** 10.15171/jrip.2016.01

**Published:** 2016-01-18

**Authors:** Wisit Cheungpasitporn, Charat Thongprayoon, Kianoush Kashani

**Affiliations:** ^1^Division of Nephrology and Hypertension, Department of Internal Medicine, Mayo Clinic, Rochester, MN, USA; ^2^Department of Internal Medicine, Bassett Medical Center, Cooperstown, NY, USA; ^3^Division of Pulmonary and Critical Care Medicine, Department of Internal Medicine, Mayo Clinic, Rochester, MN, USA

**Keywords:** Acute Kidney Injury, Chronic kidney disease, Dialysis, Transcatheter aortic valve implantation, Transcatheter aortic valve replacement, Transplantation

## Abstract

Transcatheter aortic valve replacement (TAVR) has now emerged as a viable treatment option for high-risk patients with severe aortic stenosis (AS) who are not suitable candidates for surgical aortic valve replacement (SAVR). Despite encouraging published outcomes, acute kidney injury (AKI) is common and lowers the survival of patients after TAVR. The pathogenesis of AKI after TAVR is multifactorial including TAVR specific factors such as the use of contrast agents, hypotension during rapid pacing, and embolization; preventive measures may include pre-procedural hydration, limitation of contrast dye exposure, and avoidance of intraprocedural hypotension. In recent years, the number of TAVR performed worldwide has been increasing, as well as published data on renal perspectives of TAVR including AKI, chronic kidney disease, end-stage kidney disease, and kidney transplantation. This review aims to present the current literature on the nephrology aspects of TAVR, ultimately to improve the patients’ quality of care and outcomes.

**Implication for health policy/practice/research/medical education:**

Transcatheter aortic valve replacement (TAVR) has now emerged as a viable treatment option for high-risk patients with severe aortic stenosis (AS) who are not suitable candidates for surgical aortic valve replacement (SAVR). Despite encouraging published outcomes, acute kidney injury (AKI) after TAVR is common and lowers the survival of patients after TAVR. In recent years, the number of TAVR performed worldwide have been increasing as well as published data on renal perspectives of TAVR including AKI, chronic kidney disease, end-stage kidney disease and kidney transplantation. We present the current literature on nephrology aspects of TAVR, ultimately to improve the patients’ quality of care and outcomes.

## Introduction

Aortic stenosis (AS) is one of the most common cardiac degenerative valvular diseases, with a prevalence of 1.3% in patients between 65 and 74 years and 2.8%-4.6% in patients >75 years of age (1-3). Due to an aging population, the incidence of AS continues to rise over time, and thus, AS has become a significant healthcare burden (1,3,4). Without treatment, these patients have a poor prognosis with 50% mortality in the first two years after diagnosis (5).

Surgical aortic valve replacement (SAVR) is currently considered the gold standard treatment for severe symptomatic AS (6). Transcatheter aortic valve replacement (TAVR), also known as transcatheter aortic valve implantation (TAVI), being performed since 2002, has now emerged as a viable treatment option for high-risk patients with severe AS who are not suitable candidates for SAVR (6-9). Recently published results of the 5-year outcomes from multicenter, randomized controlled trials (RCTs) demonstrated a survival benefit of TAVR over standard treatment for patients with inoperable AS (10) and comparable survival rates in high-risk patients with AS undergoing TAVR compared to SAVR (11). The applications of TAVR are also expanding to ‘off-label’ indications in patients with intermediate risk, AS secondary to bicuspid valve disease, aortic regurgitation, aortic valve-in-valve procedures, and mitral valve interventions (12). To date over 200 000 procedures have been performed worldwide. Despite the encouraging reports, post-procedural acute kidney injury (AKI) remains a common complication of TAVR, particularly when carried out in patients with high comorbidities (13,14).

## Materials and methods

This review article discusses kidney related aspects of TAVR including the incidence, predictors, and the impact of AKI following TAVR. We report the available evidence of clinical outcomes of patients with chronic kidney disease (CKD), end-stage renal disease (ESRD), and renal transplantation who undergo TAVR.

For this review, we used a variety of sources by searching through PubMed, EMBASE, Scopus and directory of open access journals (DOAJ). The search was performed using combinations of the following key words and or their equivalents; acute kidney injury, chronic kidney disease, dialysis, transcatheter aortic valve implantation, transcatheter aortic valve replacement and transplantation.

## AKI after TAVR

### Incidence of AKI After TAVR

Due to many different definitions of AKI used in the literature (15,16), the reported incidence of AKI after TAVR varies widely (13,14). By using a consensus AKI definition (modified RIFLE) (17), the reported incidence of AKI following TAVR ranged from 15% to 57%, with the need for renal replacement therapy (RRT) in 2%-40% of all patients (6,13,14,18-24). Compared to patients without AKI, patients who developed AKI after TAVR had a higher mortality rate of 9%-44% at 30 days and 32%-56% at 1 year (13,14).

In 2012, the Valve Academic Research Consortium (VARC) published their updated endpoint definitions in the VARC-2 consensus (25) recommending standardized criteria, i.e., the Acute Kidney Injury Network (AKIN) criteria, and most recently, the Kidney Disease Improving Global Outcomes (KDIGO) criteria (26). In addition, VARC-2 standardized the timing for the AKI diagnosis, extending from 72 hours to 7 days following a TAVR procedure. With these standardized criteria, we recently reported the incidence of AKI within 7 days following TAVR of 28% (22% in stage 1, 2% in stage 2, and 4% in stage 3) and the need for RRT during hospitalization of 3% (27).

### Pathophysiology and risk factors of AKI after TAVR

Similar to SAVR-associated AKI, the pathogenesis of TAVR-related AKI is multifactorial including perioperative renal hypoperfusion related to a combination of pre-, intra- and postoperative factors (Table 1) (13,14,28). Although the use of cardiopulmonary bypass during TAVR is not required, TAVR itself is associated with specific AKI risks. Catheter-based techniques and valve implantation, use of contrast agents, hypotension during rapid ventricular pacing for balloon valvuloplasty and valve deployment, and embolization resulting from the manipulation of catheters in the aorta of patients with diffuse atherosclerosis are examples of intraoperative risk factors for AKI (28).

**Table 1:** Reported potential predictors/perioperative factors associated with postoperative AKI following TAVR^a^

**Preoperative**	**Intraoperative**	**Postoperative**
-Older age -Pre-existing chronic kidney disease -Short-interval contrast exposure -Congestive heart failure -Peripheral Vascular Disease-Diabetes -Logistic EuroSCORE	-Periprocedural Bleeding and Blood Transfusion -Embolic events-Contrast agents -Hypotension from rapid ventricular pacing -Transapical approach -Complicated cases requiring intra-aortic balloon pump	-Vasoconstricting agents Nephrotoxins -Decreased heart function -Hemodynamic instability -Grade of aortic regurgitation after the procedure

Abbreviations: AKI, acute kidney injury; TAVR, Transcatheter Aortic Valve Replacement. ^a^References 13, 14, 28, and 35.

### Transapical approach and cholesterol emboli

The TAVR procedure can be performed through a few approaches such as transfemoral (TF), transapical (TA) and transaortic routes. There are a few advantages to using TF-TAVR over other techniques, including the ability to use moderate sedation and local anesthetics and shorter procedure and recovery times (29). Therefore, at most centers the TF-TAVR strategy is considered first (13,14,28,29). However, not all patients are suitable for TF-TAVR due to advanced peripheral vascular disease; in these cases, very small or severely calcified or tortuous peripheral vessels can preclude safe placement of the access sheath (28,29). For these patients, TA-TAVR is the method of choice (30). Unfortunately, several studies have demonstrated an association between the TA approach and higher risk of AKI (31-33). The mechanisms of observed higher AKI risk are only speculative, but a possible explanation could be the difference in patient populations. Those undergoing a TA-TAVR have more severe peripheral vascular atherosclerotic disease, which is per se a risk factor for AKI after TAVR (Table 1) (34,35). In addition, the aortas of patients with severe peripheral vascular disease are usually more atherosclerotic. During the TA-TAVR procedure, the instrumentation of the aorta may result in the dislodgement of calcium plaques and cholesterol emboli to the renal vascular bed, leading to AKI (35). Also, as discussed earlier, TA-TAVR is mostly performed under general anesthesia, which could be associated with general and renal hypoperfusion, while TF-TAVR is performed under moderate sedation and local anesthetics and is commonly not associated with hemodynamic instability (29).

## Contrast agent exposure

Despite advances in TAVR technique, the TAVR procedure still requires fluoroscopy and angiography using contrast agent to aid in positioning the valve (36), which may result in contrast-induced AKI (CIAKI). However, the impact of contrast agent utilization on AKI after TAVR remains controversial. A few studies suggest an association between contrast media and higher AKI incidence following TAVR (37,38), especially in patients with pre-existing CKD (39). However, other reports have not demonstrated such association (32,34,40-43). Minimization of the contrast dose during TAVR to <100 mL and use of low or iso-osmolar contrast media can explain these observations (34,35,37,44).

### Hypotension from rapid ventricular pacing

During the TAVR procedure, the positioning of balloon-expandable valves requires rapid ventricular pacing via a right ventricular temporary pacing wire to reduce aortic pressure and achieve cardiac standstill (14,29). It has been suggested that excessive hypotension, induced by rapid ventricular pacing, may theoretically lead to a decrease in renal perfusion and renal ischemia-reperfusion injury, with an increased risk of AKI. Interestingly, Bagur et al (41) evaluated the number of procedural rapid pacing runs in the development of AKI following TAVR and found no significant correlation. Despite this report, the impact of the rapid pacing duration on AKI following TAVR requires more detailed investigation (35,41).

## AKI after transcatheter or surgical aortic valve replacement

Among studies that compared the incidence of postoperative AKI events in patients with severe AS undergoing TAVR versus SAVR (13), several studies showed a higher incidence of AKI among patients who underwent TAVR (23,45). However, it should be noted that patients selected for TAVR typically have higher comorbidities which may carry a higher risk for AKI. Therefore, we recently conducted a meta-analysis of 3 randomized control trials with a total of 1852 patients and 14 cohort studies with 3113 patients and found a lower AKI risk among TAVR patients when compared with SAVR (13).

It is unclear whether the different study outcomes were due to patient risk profiles confounding the results, or whether the heterogeneity of AKI definitions among included studies were the cause. Thus, we undertook a study of 1563 adult patients undergoing isolated TAVR or SAVR for severe AS at Mayo Clinic Hospital in Rochester, Minnesota from January 1, 2008, to June 30, 2014 (27). We performed a propensity-matched comparison for the postoperative incidence of KDIGO-defined AKI within 7 days of the procedure as recommended by the VARC-2 consensus (25). Among the 195 matched pairs (390 patients), baseline characteristics, including Society of Thoracic Surgeons (STS) risk score and estimated glomerular filtration rate eGFR, were comparable between the two groups. We found no significant difference in postoperative AKI incidence (24.1% versus 29.7%; *P*=0.21) between the TAVR and SAVR groups. In addition, there were no differences in major adverse kidney events, the composite of in-hospital death, use of RRT during hospitalization and persistence of renal dysfunction at hospital discharge (2.1% versus 1.5%; *P*=0.70), or mortality >6 months after surgery (6.0% versus 8.3%; *P*=0.51) (27).Thus, TAVR did not affect postoperative AKI risk and may be preferred in high-risk patients with severe AS considering its less invasive nature compared to SAVR.

## TAVR in CKD

Based on the data from the Edwards SAPIEN Aortic Bioprosthesis European Outcome (SOURCE) Registry, CKD is considered one of the strongest independent predictors of 1-year mortality following TAVR (46). It is well established that patients with CKD carry a higher risk of AKI (47).Interestingly, in the setting of AKI after TAVR, studies have demonstrated different findings regarding the impact of CKD on AKI occurrence. Elhmidi et al (40) studied 234 patients with severe AS who underwent TAVR between 2007 and 2010 at a single center and found that preoperative serum creatinine level was the only independent predictor of postoperative AKI. Khawaja et al (48)*,* subsequently demonstrated that higher CKD stage had the strongest independent associations with AKI after TAVR. However, a number of studies did not detect an association between CKD and AKI following TAVR (31,34,41,49).

Voigtländer et al (50), recently studied the influence of kidney function before TAVR on the AKI incidence in 540 patients. Investigators divided patients into three groups according to their GFR before TAVR (GFR ≥60 (normal renal function), 30–59 (moderate impaired renal function), and <30 mL/min/1.73 m^2^[severe impaired renal function]). Overall, there was an increase in GFR after TAVR from baseline GFR of 59.1±21.7 ml/min/1.73 m^2^ to 63.6±23.6 ml/min/1.73 m^2^ at hospital discharge. The investigators demonstrated a modest increase in GFR in the moderately impaired renal function group and a significant increase in GFR in those with severe decreased renal function. There was no significant change in GFR after TAVR in patients with normal renal function (50). The improvement of GFR was also demonstrated at one month following TAVR in patients with preexisting CKD (51). It is therefore possible that renal function improved in some cases following TAVR due to the improvement of cardiac performance following correction of valvular disease (28).

## TAVR in ESRD

Valvular heart disease, and especially AS, is more prevalent in patients with ESRD undergoing maintenance dialysis than the general population due to calcification of the aortic valve associated with secondary hyperparathyroidism (52). In ESRD patients, aortic valve calcification occurs 10-20 years earlier and progresses more rapidly than in the general population (53). In addition, ESRD patients often carry multiple comorbidities and bear a high risk of complications following SAVR. On the other hand, the mortality rate is higher and quality of life in lower when ESRD patients have symptomatic AS (53). Recently, Kobrin et al (54) studied all Medicare fee-for-service patients (5005 undergoing TAVR and 32634 undergoing SAVR) between January 1, 2011, and November 30, 2012. Compared to non-dialysis patients, TAVR patients on dialysis had a significantly higher rate of mortality at 30 days (13% vs. 6%) and lower survival at one year (57.4% vs. 77.4%). In the propensity-matched comparison of 194 matched pairs of dialysis SAVR and dialysis TAVR patients (388 patients), the investigators reported shorter length of hospital stay with comparable survival in TAVR patients receiving dialysis (54). Thus, TAVR potentially plays a significant role in patients with ESRD and severe AS.

## TAVR in kidney transplant recipients

During kidney transplant candidate evaluation, screening for AS with history, physical examination, and echocardiogram is recommended for all patients if clinical suspicion for AS is high (55,56). It is recommended that patients with moderate to severe AS be considered for valve replacement before kidney transplantation. Even after transplantation, cardiovascular and valvular diseases are still prevalent and remain one of the leading causes of death in kidney transplant recipients (57). Furthermore, the incidence of AS among kidney transplant patients will likely rise as their survival improves and as the mean age of patients undergoing kidney transplantation increases (58). Data on the survival of kidney transplantation patients after cardiac valve replacement are limited. Recent data from the US Renal Data System database demonstrated mortality rates of kidney transplant recipients undergoing valvular heart surgery of 14% in the hospital and 40% within 2 years of surgery (57). In addition, kidney transplant recipients with severe AS are often found unsuitable for SAVR due to impaired kidney function, possible side effects of immunosuppressive medication, and comorbidities (58).

Fox et al (58) reviewed the outcomes of eight kidney transplant recipients with severe AS undergoing TAVR (6 transfemoral; 2 transapical). The investigators reported that all TAVR procedures were performed successfully with excellent functional results. After TAVR, all kidney transplant recipients were alive at the 12-month follow-up with only one reported cardiovascular event. Despite encouraging outcomes after TAVR in kidney transplant recipients, aortic root rupture, a rare but fatal complication of TAVR, was recently reported in two renal transplant patients (59). However, it is still unclear if chronic immunosuppressant therapy is associated with aortic root rupture, and future studies are needed for transplant recipients undergoing TAVR.

## Measures to prevent AKI after TAVR

As discussed earlier in this review, the pathogenesis of AKI after TAVR is multifactorial. Measures to prevent AKI after TAVR are proposed in Table 2. Although the data on the impact of contrast exposure on the incidence of AKI after TAVR are still controversial, avoiding repeated exposure to contrast dye over a short period, minimizing the volume of contrast agent, especially in patients with CKD, and careful hydration based on the individual’s cardiac performance should be considered. Intraprocedural hypotension should be avoided in all cases. Patients who undergo TAVR with a transapical approach, patients with renal insufficiency, or patients requiring an intra-aortic balloon pump use should be considered at risk for developing AKI after TAVR, and preventive measures including preprocedural hydration, limitation of potentially nephrotoxic agents, and a judicious use of blood transfusions should be considered.

**Table 2:** Measures to prevent acute kidney injury after transcatheter aortic valve replacement

**General measures**
Minimize repeated exposure to contrast dye during a short period
Minimize the volume of contrast agent
Avoid intraprocedural hypotension
Avoid bleeding and restrict the use of blood transfusions
**Potential future preventive measures**
RenalGuard system (Forced diuresis with matched hydration)
Doppler-based renal resistance index top predict risk for AKI
TAVR Embolic Protection Systems to prevent renal embolism

Abbreviations: AKI, acute kidney injury; TAVR, transcatheter aortic valve replacement.

The RenalGuard system, a dedicated device with forced diuresis and matched hydration designed for CIAKI, has recently been introduced in the TAVR setting and appears to be safe and efficient (60). Although investigators demonstrated a smaller creatinine rise in a RenalGaurd group compared to a standard of care cohort, they did not report differences in important clinical endpoints, including rate of RRT or mortality (60,61). While the attempts to reduce stroke rates by TAVR embolic protection systems are promising (62), future studies on intervention or preventive measures to prevent renal emboli, especially in patients with CKD, are needed. Another intervention which requires further validation is the use of a Doppler-based renal resistance index to predict and identify patients at risk for AKI (63). In the future, progress in the research of urine biomarkers (64), electronic health records with an AKI alert (65) for early AKI detection, and risk stratification models will likely improve the renal outcomes of patients undergoing TAVR.

## Conclusion

The prevalence of AS continues to rise over time among our aging population. With advances in the field of interventional cardiology, TAVR has already become a treatment option for severe AS in the inoperable or high-risk surgical candidate. In addition, the use of TAVR for the treatment of other pathologies and lower-risk patients is being explored. The number of TAVR procedures will continue to increase in the general patient population as well as in patients with CKD, ESRD, and in candidates for kidney transplantation. Thus, physicians should understand and be aware of potential complications following the TAVR procedure. The occurrence of AKI following TAVR is common and a prognostically significant complication. The ultimate success of TAVR depends on careful attention to detail and prompt management of complications and understanding the risk factors for AKI after TAVR. Growing knowledge of the potential impact of TAVR on kidney function will help improve patient selection, TAVR technique, and preventive measures to improve patients’ outcomes.

## Authors’ contribution

WC, CT, and KK contributed to the manuscript equally.

## Conflicts of interest

The authors declare that they have no conflicting interest.

## Ethical considerations

Ethical issues (including plagiarism, data fabrication, double publication) have been completely observed by the authors.

## Funding/Support

None.
